# Design and Optimization of a Hyper-Branched Polyimide Proton Exchange Membrane with Ultra-High Methanol-Permeation Resistivity for Direct Methanol Fuel Cells Applications

**DOI:** 10.3390/polym10101175

**Published:** 2018-10-22

**Authors:** Liying Ma, Guoxiao Xu, Shuai Li, Jiao Ma, Jing Li, Weiwei Cai

**Affiliations:** 1 School of Chemistry and Materials Science, Guizhou Normal University, 116 Baoshan North Road, Guiyang 550001, China; maliying2018@163.com (L.M.); Mj68031104@163.com (J.M.); 2 Sustainable Energy Laboratory, Faculty of Materials Science and Chemistry, China University of Geosciences Wuhan, 388 Lumo RD, Wuhan 430074, China; guoxiao_xu123@163.com (G.X.); lishuai19970115@163.com (S.L.)

**Keywords:** proton exchange membrane, direct methanol fuel cell, methanol resistivity, composite membrane

## Abstract

A hyper-branched sulfonated polyimide (*s*-PI) was synthesized successfully and composited with polyvinylidene fluoride (PVDF) to achieve ultra-high methanol-permeation resistive for direct methanol fuel cell application. The optimized *s*-PI-PVDF composite membrane exhibited methanol resistivity low to 1.80 × 10^−8^ cm^2^/s, two orders of magnitude lower than the value of the commercial Nafion 117 membrane (60 × 10^−7^ cm^2^/s). At the same time, the tensile strength of the composite membrane is 22 MPa, which is comparable to the value of the Nafion 117 membrane. Therefore, the composite membrane is promising for application in direct methanol fuel cell.

## 1. Introduction

Recently, polymer exchange membrane fuel cells (PEMFCs) have attached increasing attention for applications such as low-emission vehicles, stationary power and portable electronics [1,2]. The key component of PEMFC is the proton exchange membrane (PEM), which conduct protons and separate the fuel from oxidant. The important role of an efficient PEM contain high proton conductivity, good mechanical, chemical, thermal and dimensional stability, and low fuel permeability [3,4,5,6]. A polyperfluorosulfonic acid proton membrane nafion (Dupont) is the most widely used PEM in PEMFCs due to the high proton conductivity and good chemical stability [7,8]. However, the high cost and especially the serious methanol permeation of Nafion hinder the potential application in Direct Methanol Fuel Cells (DMFCs) [9,10]. As an alternative to Nafion, partially fluorinated [11,12,13,14,15,16,17] and non-fluorinated PEMs [18,19,20,21,22,23,24,25,26,27,28,29] have been widely developed.

Among many advanced sulfonated PEMs, such as poly (ether ether ketone) (PEEK) [30,31], poly (ether ketone) [32], polyamide [33,34,35,36,37], poly (arylene ether) [38,39] and polyimide [2,40,41], sulfonated polyimide is considered one of the most potential alternative PEM to be used in fuel cells [41]. Watanabe et al. [40] synthesized several sulfonated polyimide copolymers and showed good oxidative stability, mechanical strength and proton conductivity. Ding et al. [41] prepared a series of sulfonated polyimides proton exchange membranes; the membranes exhibit acceptable proton conductivity and low methanol permeability.

In this study, we designed and synthesized a hyper-branched non-fluorinated sulfonated polyimide (*s*-PI) based composite PEM to overcome the methanol crossover issue. In order to enhance the mechanical strength of the PEMs, polyvinylidene fluoride (PVDF) was used as a binder due to the great mechanical stability and high flexibility [42]. Structural and thermodynamic compatibility, which was proved to be of great importance to the quality of the blend membrane [43,44,45], between PVDF and PI has been proved [46]. The main properties of the PEMs, including the proton conductivity, methanol permeability and mechanical/thermal property, thermal stabilities were systematically investigated. The optimized PEM exhibits a methanol permeability that is low to 1.9 × 10^−8^ cm^2^/s, more than two orders of magnitude lower than that of the commercial Nafion 117 membrane [47]. In addition, the tensile stress of the optimized PEM is as high as 22 MPa. The overall performances suggest that the *s*-PI based composite PEM has the potential of application in DMFC.

## 2. Experimental

### 2.1. Materials

Phloroglucinol (PL), 4-nitrophthalontrile (NPN), 2,4-diaminobenzenesulfonic acid (DSA), m-cresol and polyvinylidene fluoride (PVDF) were provided by Sigma-Aldrich Reagent, Ltd. (St. Louis, MO, USA), and dried for 24 h at 80 °C before using. Dimethyl sulfoxide (DMF), (CH_2_OH)_2_, K_2_CO_3_, KOH and HAc were purchased from Sinopharm Chemical Reagent Co., Ltd. (Chengdu, China)

### 2.2. Synthesis

**Scheme 1:**
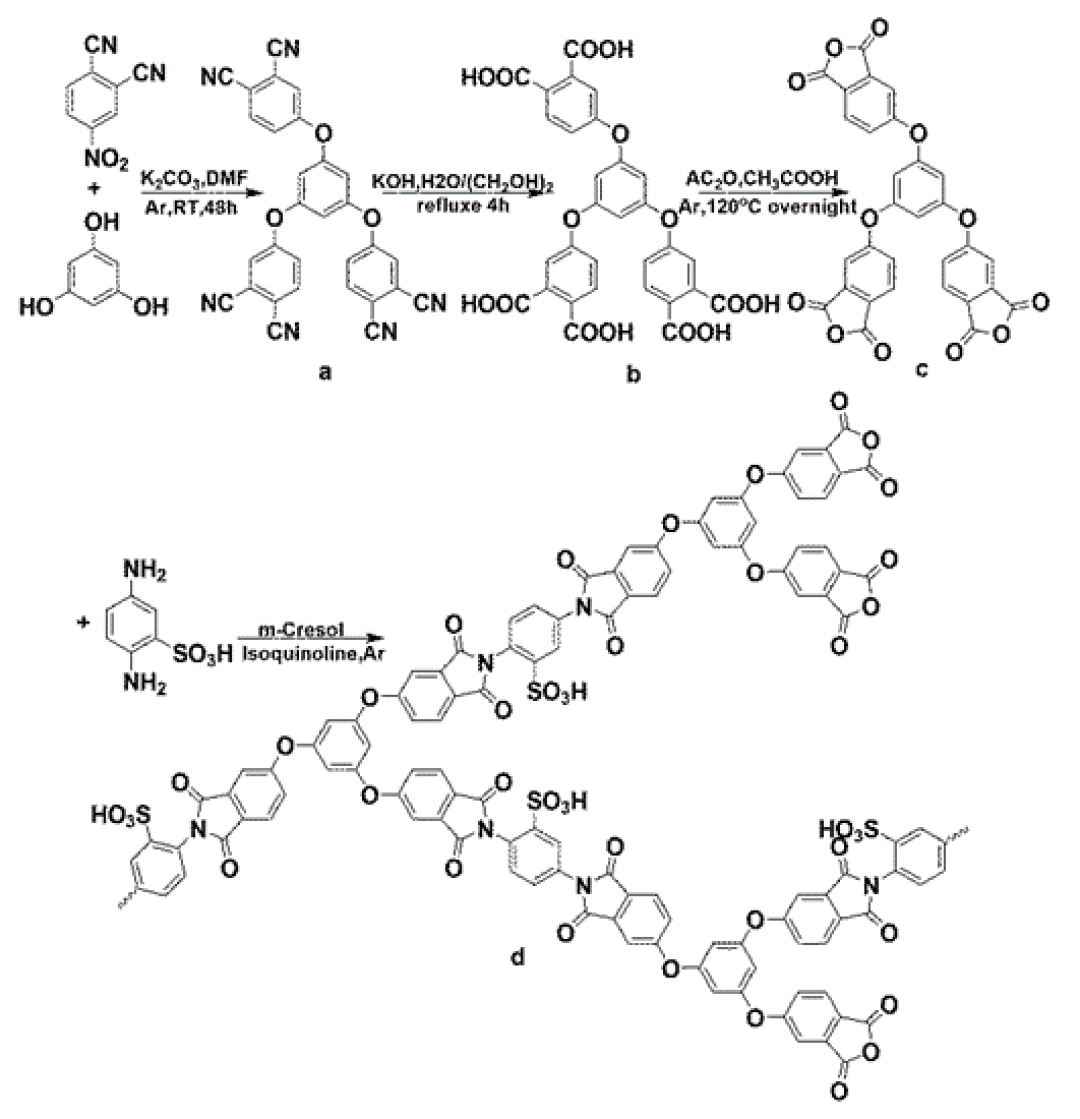
Route of polymer synthesis.

Synthesis of product a: PL (10 mmol), NPN (30 mol), K_2_CO_3_(8.28 g), and DMF (60 mL) were added in to a 100 mL two-necked round-bottom flask, the reaction was maintained 25°C for 48 h under argon atmosphere. Upon completion, the mixture was poured into 500 mL water, filtered and washed with water, and then dried at 80 °C for 24 h (Scheme 1).

Synthesis of product b: Product a (4.5 mmol), KOH (2.77 g), H_2_O (12 mL) and (CH_2_OH)_2_ (12 mL) were added into a 100 mL round-bottom flask, the mixture was kept stirred at 115 °C for 4 h under argon atmosphere. After cooling to RT, the resulting solution was filtered and washed with water several times, and the white crystal was dried at 80 °C for 24 h (Scheme 1).

Synthesis of product c: Product b (2 mmol), HAc (20 mL), Ac_2_O (2 mL) were added in to 50 mL round-bottom flask, the reaction was maintained at 120 °C overnight. After the reaction was completed, the resulting solution was cooled to RT, then filtered and washed with cold water. The white products were dried under vacuum at 80 °C for 24 h (Scheme 1).

Synthesis of polymer *s*-PI (d): Product c (5 mmol), DSA (5 mmol), m-cresol (5 mL) and isoquinoline (0.2 mL) were added in to a 50 mL round-bottom flask, the reaction was kept stirred at 100 °C for 5 h under argon atmosphere and then rose to 120 °C overnight. The solution was poured into 50 mL methanol, then filtered and washed with methanol, and dried at 80 °C for 24 h under vacuum (Scheme 1).

### 2.3. Membrane Preparation

The membranes were fabricated via a solution casting technique. The hybrid homogeneous solution of polymer and PVDF in dimethyl sulfoxide (DMSO) were filtered and cast on clean glass plates and dried at 80 °C. All the dried membranes were treated in 1M HCl solution for 12 h at room temperature, and then washed with deionized water several times until the pH = 7.

### 2.4. Measurements and Characterizations

^1^H nuclear magnetic resonance (NMR) spectra of the synthesized products and polymer were recorded using a Bruker AVANCE IIIHD instrument (400 MHz, Bruker, Hamburg, Germany) in deuterated dimethyl sulfoxide (DMSO-d_6_) as solvent. The FTIR spectra of synthesized products and polymer were conducted on a FTIR Nicolet6700 spectrometer (Thermo Fisher Scientific, Waltham, MA, USA). Thermogravimetric (TG) analysis of the composite membranes was recorded on a NETZSCH simultaneous thermal analyzer (Netzsch, Bavaria, Germany) at 10 °C /min ramping up to 600 °C under a nitrogen atmosphere. Cross-section morphologies of the composite membranes were obtained from a field emission scanning electron microscopy (SEM, SU8010, Hitachi, Tokyo, Japan). Proton conductivity was measured by four-electrode AC impendence method with Interface 1000 electrochemical workstation (Gamry, Philadelphia, PA, USA). Proton conductivity was measured under the temperature from 25 to 80 °C with a relative humidity of RH = 100%. Proton conductivity was calculated using the following Equation:

(1) $$\sigma =\frac{d}{Rtw}\;$$

where *σ* is the proton conductivity of the membranes (S/cm), *w* and *t* refer to the width (cm) and thickness (cm) of the membrane, respectively. *d* stands for the distance between the two electrodes (cm). *R* is the resistance of the measured impedance data (Ω).

The methanol permeability of composite membranes was carried out via an “H” type diaphragm diffusion cell. One diffusion cell contained 10.0 M methanol solution and the other sampling cell contained the same volume of deionized water, which was separated by the membrane and stirred with magnetic stirrer for diffusion. The concentration of methanol in the sampling cell was measured via a gas chromatograph (9790II, Zhejiang Fuli Analytical Instruments Co., Ltd., Wenling, China). The methanol permeability was calculated according to the Equation as follows:

(2) $$p=\frac{l}{A}\times \frac{V_{B}}{C_{A}}\times \frac{\Delta C}{\Delta t}\;$$

where *p* is the methanol permeability (cm^2^/s), *C*_A_ is the initial concentration of methanol (mol/cm^3^), *V*_B_ is the volume of the compartment (cm^3^), *A* and *l* are the effective diffusion area and the thickness, respectively. Δ*t* and Δ*C* are diffusion time (s) and methanol concentration (mol/cm^3^), respectively.

Ion exchange capacity (*IEC*) of the composite membranes was measured through the classical acid-base titration method. Dried membrane was first immersed in 1 M NaCl solution for 48 h at room temperature to replace the H^+^ ions with Na^+^ ions, and then the content of exchange proton H^+^ was titrated with 0.01 M NaOH solution using phenolphthalein as an indicator. The *IEC* value was calculated according to the following Equation:

(3) $$IEC=\frac{V\times C}{W}\left(\mathrm{mmol}/g\right)\;$$

where *V* and *C* represent for the volume (mL) and the concentration (mol/L) of NaOH solution, respectively. *W* stands for the weight of dry membrane (g).

Water uptake and volume swelling were tested according to the following process. A piece of composite membrane sample was first placed in ultrapure water for 12 h at an ambient temperature, then the weight and the volume were immediately recorded upon removal of the surface water with tissue paper. The membrane was dried at 80 °C for 12 h under vacuum to remove the residual water to measure the volume and the weight quickly. Water uptake (*WU*) and volume swelling (*VS*) were obtained using the below Equation, respectively:

(4) $$WU=\frac{\left(W_{\mathrm{Wet}}-W_{\mathrm{dry}}\right)}{W_{\mathrm{dry}}}\times 100\%\;$$

where *W*_wet_ and *W*_dry_ are the weight of wet and dry membranes, respectively.

(5) $$VS=\frac{V_{\mathrm{Wet}}-V_{\mathrm{dry}}}{V_{\mathrm{dry}}}\times 100\%\;$$

where *V*_wet_ and *V*_dry_ are corresponding to the volume of the wet and dry membranes, respectively.

The tensile stress and elongation at break of a rectangular composite membrane samples was measured using Labthink XLW (PC) (Labthink, Jinan, China) with a 50 N load cell at a tensile speed of 25 mm/min at ambient temperature.

## 3. Results and Discussion

**Figure 1:**
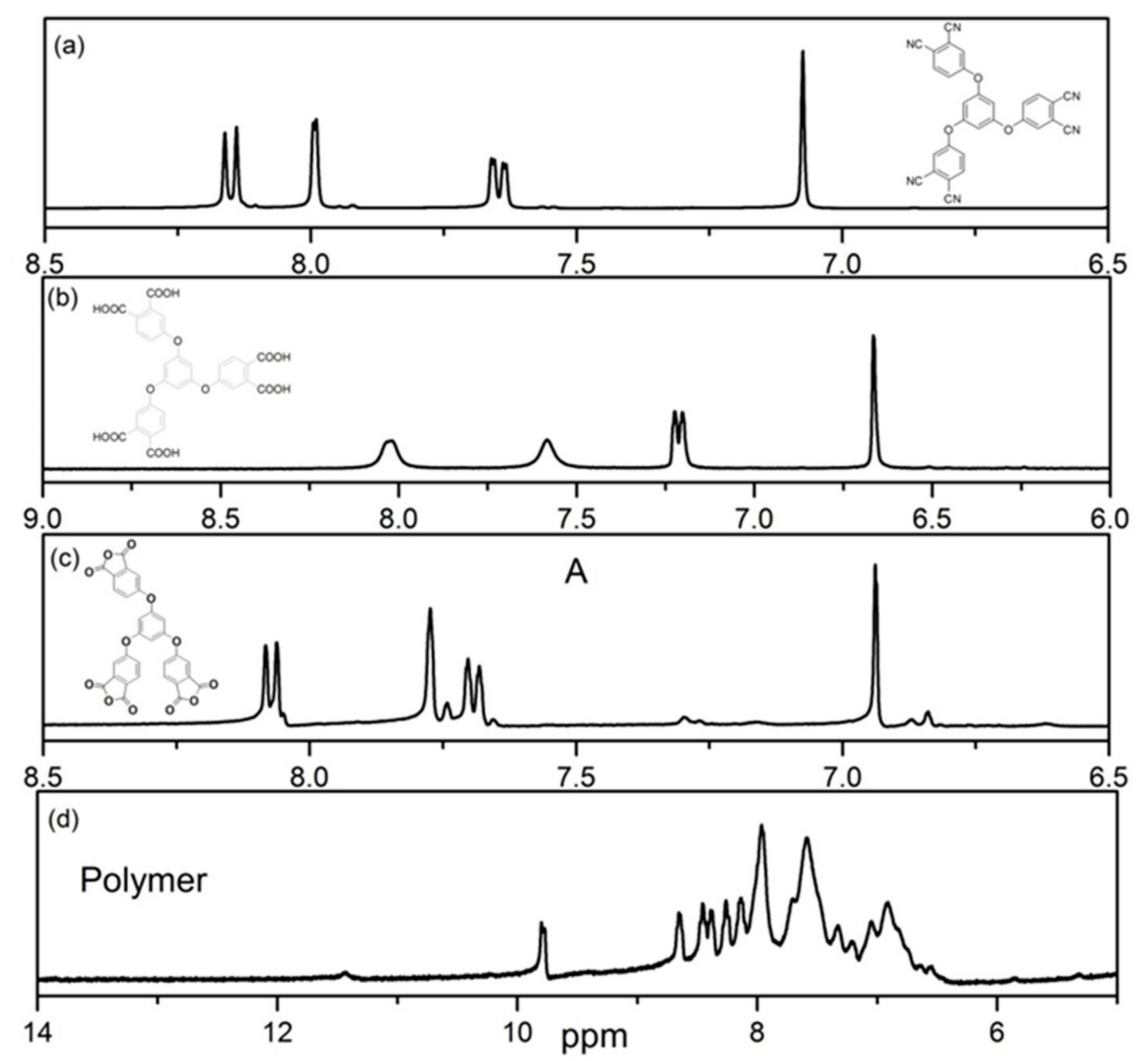
^1^H NMR spectra of the intermediate product (**a**–**c**) and the sulfonated polyimide (*s*-PI) polymer (**d**).

Figure 1 displays the ^1^H NMR spectra of the intermediate products and the *s*-PI polymer. For the intermediate product a, ^1^H NMR (400 MHz, DMSO-d_6_), the distinct resonances at 8.2, 8.0 and 7.6 ppm present the phenyl group with –CN group. The signal at 7.1 ppm is attributed to the phenyl group. For the product b, ^1^H NMR (400 MHz, DMSO-d_6_), the distinct resonances at 7.8, 7.3 and 7.1 ppm ascribe to the hydrogen atom of the phenyl group with –COOH group. The signal at 6.6 ppm is attributed to the phenyl group inside. For precursor c, ^1^H NMR (400 MHz, DMSO-d_6_), the distinct resonances at 8.1, 7.8 and 7.7 ppm ascribe to the three phenyl group outside, while the signal at 6.9 ppm is assigned to the phenyl group inside. For polymer, the signals at 11.5 ppm presented the –SO_3_H of polymer.

**Figure 2:**
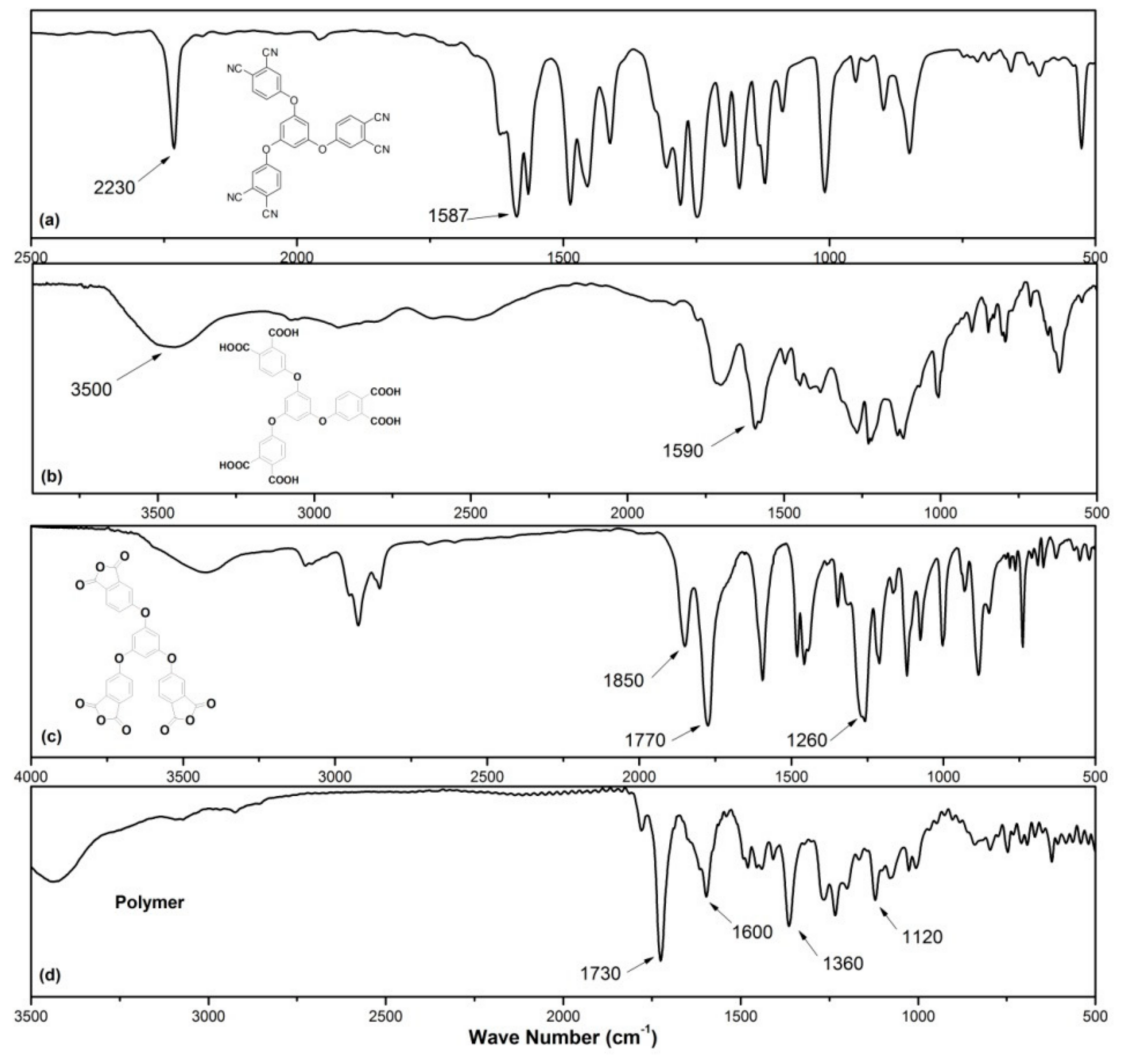
FTIR spectra of the product (**a**–**c**) and s-PI polymer (**d**).

In addition to ^1^H NMR, successful synthesis of the intermediate products and the *s*-PI polymer was confirmed by FTIR spectra provided in Figure 2. For the intermediate product a, the FTIR spectrum depicts the C≡N stretching bands at about 2230 and 1600 cm^−1^. For intermediate product b, 3500–3200 cm^−1^ (*υ*_OH_, –COOH), 1600 cm^−1^ (*υ*_C=O_, –COOH). For product c, the bands at 1850 and 1770 cm^−1^ indicate the presence of the C=O stretching vibration, and the band at 1260 cm^−1^ reflects the C–O stretching vibration of cyclic anhydride. For polymer, the peak at 1730 and 1600 cm^−1^ confirms the presence of the C=O stretching of the anhydride group, the bands of 1360 and 1232 cm^−1^ reflects the C=O stretching of the carboxylic group. The bands at 1120 cm^−1^ ascribed to the –SO_3_H group.

**Figure 3:**
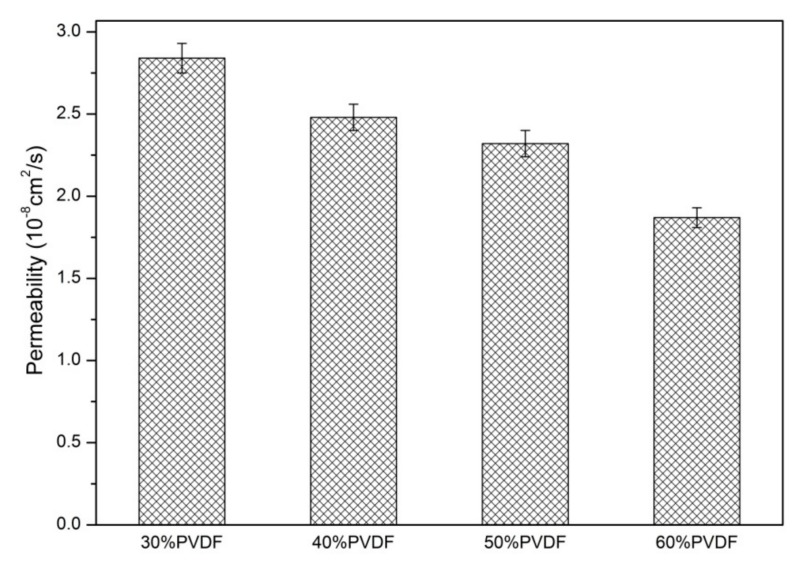
Methanol permeability through the *s*-PI-polyvinylidene fluoride (PVDF) composite membranes.

**Figure 4:**
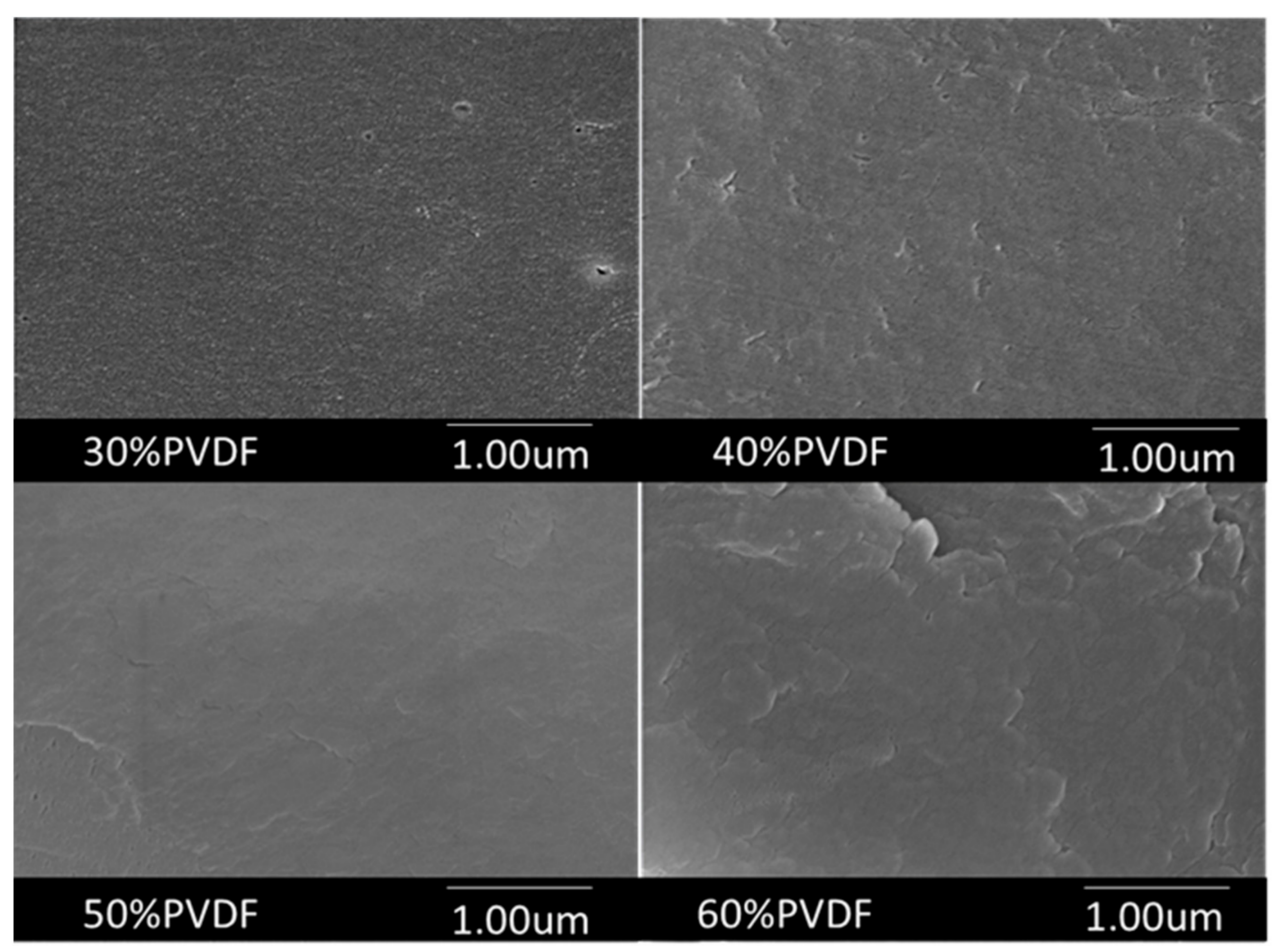
The cross-section SEM images of the *s*-PI-PVDF composite membranes.

The methanol permeation property of a PEM is the key factor when considering the DMFC application. Methanol permeability through the *s*-PI-PVDF composite membranes was depicted in Figure 3. The composite membrane described methanol permeability in the range of 1.80 × 10^−8^ cm^2^/s to 2.80 × 10^−8^ cm^2^/s, much lower than that through the Nafion 117 membrane (60 × 10^−7^ cm^2^/s) [47]. Apparently, with the PVDF weight ratio increasing from 30% to 60%, the methanol permeability value is remarkably depressed. The ultra-low methanol permeability of all the four composite membranes can also be illustrated by the flat and uniform topography SEM image of the smooth cross-section surface, as shown in Figure 4.

**Figure 5:**
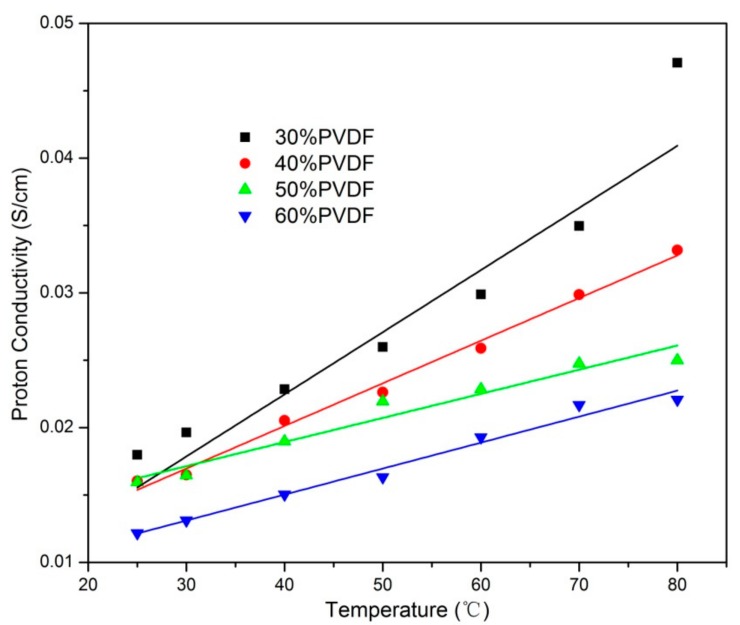
Temperature dependences of proton conductivity of the *s*-PI-PVDF composite membranes.

As one of the most important properties of any PEM, proton conductivities of the composite membranes measured at different temperatures are displayed in Figure 5. Membranes with higher *s*-PI polymer content exhibit higher conductivity due to the higher concentration of proton in the membrane contributed from the acidic sulfonamide groups while PVDF is inert on proton conduction. At the same time, proton conductivity increases with the increased temperature for all the measured membranes due to the depended of proton mobility on temperature. Unfortunately, the proton conductivity of the composite membranes is still much lower than that of the Nafion 117 (80 °C, 0.19 S/cm). It can be found that with 30% PVDF hybrid, the composite membrane exhibited the best proton conductive property among the four membranes. The trend of proton conductivity also agrees with that of the measured *IEC* value of the membranes as listed in Table 1. With the *s*-PI polymer content in membrane increased from 40% to 70%, *IEC* value of the composite membranes increased from 0.35 to 0.62 mmol/g, which is also much lower than that of the Nafion 117 membrane.

**Figure 6:**
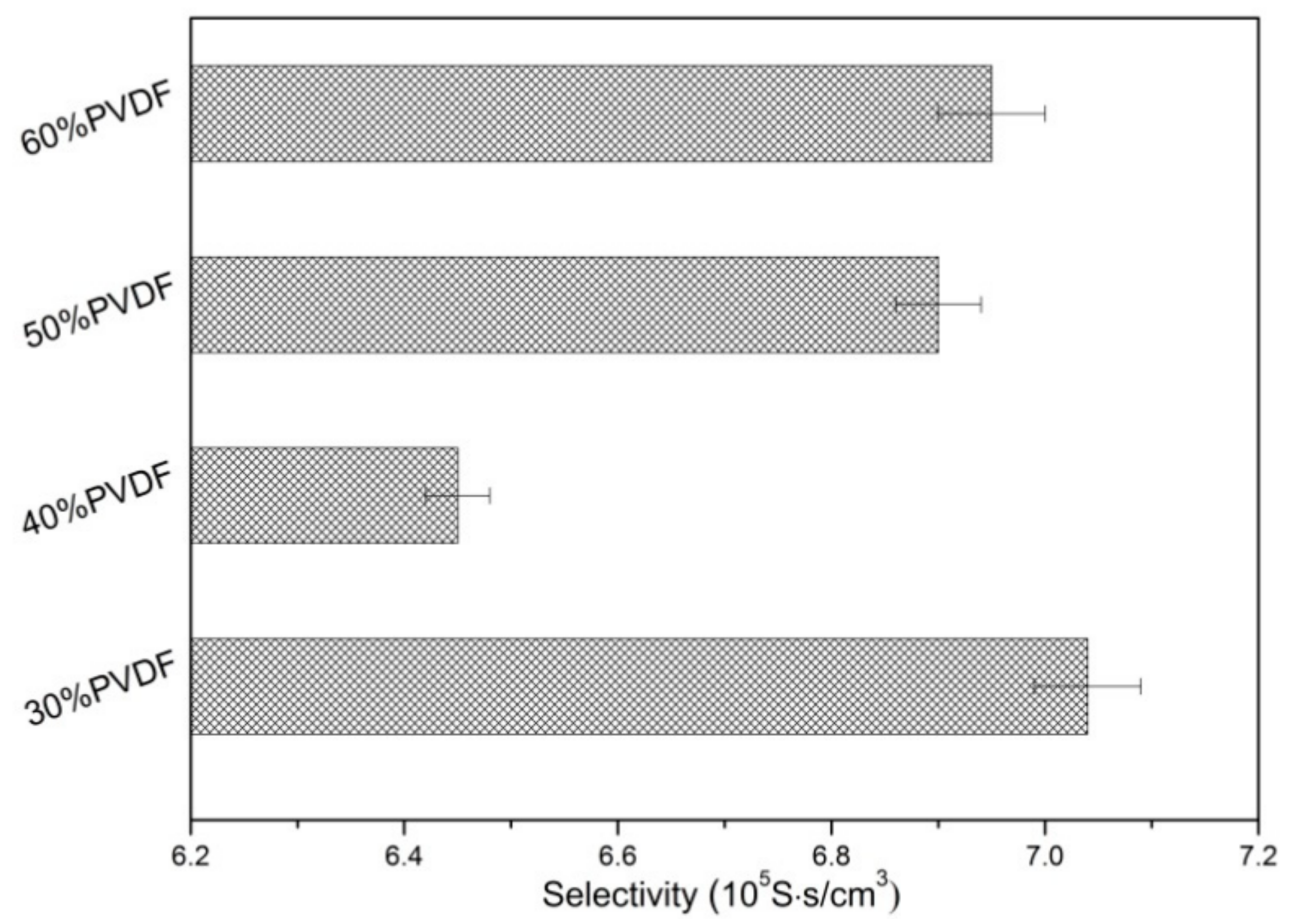
Selectivity of the *s*-PI-PVDF composite membranes.

To evaluate the overall performance of the *s*-PI-PVDF composite membrane for DMFC application, the selectivity *s* = *σ*/*p*, where *σ* is the proton conductivity and *p* is the methanol permeability, of the *s*-PI-PVDF membranes were calculated as compared in Figure 6. It can be found that the membrane with 30% PVDF content exhibited the greatest selectivity of higher than 7 × 10^5^ Ss/cm^3^. As a result, 30% is considered as the optimal PVDF content in the *s*-PI-PVDF composite membrane for DMFC application.

Water retention capacity of the PEM is one of the key factors due to water serves as the proton carrier during proton transporting according to vehicle mechanism [32,48] while excessive water would diminish the mechanical stability of the membrane [49]. The water uptake and volume swelling ratio of the *s*-PI-PVDF composite membranes were measured and are depicted in Table 1. All of the *s*-PI-PVDF composite membranes exhibit lower water uptake and volume swelling than that of Nafion 117 do. It verifies that the *s*-PI-PVDF composite membrane expresses much lower methanol permeability than that of the Nafion 117, due to the high water uptake commonly leading to the generation of methanol crossover channels.

**Figure 7:**
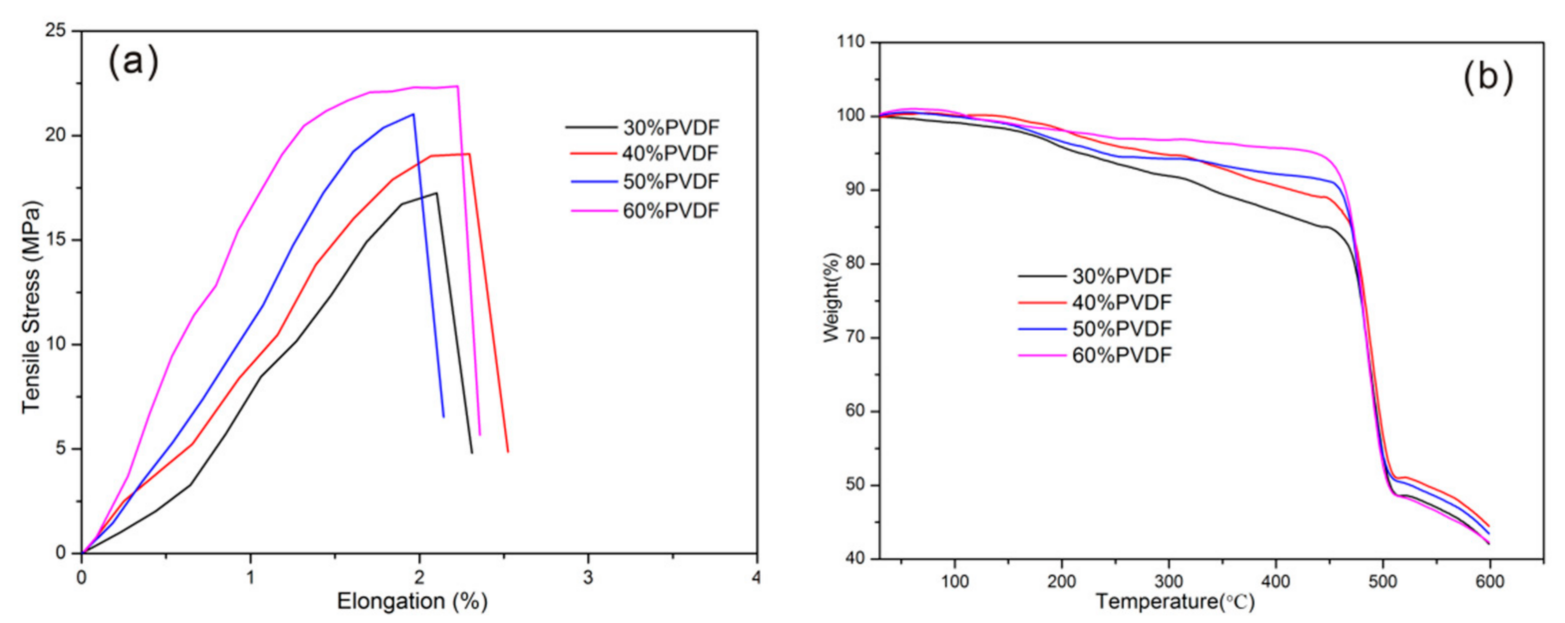
The stress-strain relationship (**a**) and the thermogravimetric (TG) curves (**b**) of the *s*-PI-PVDF composite membranes.

Mechanical stability and thermal stability of the *s*-PI-PVDF composite membranes were subsequently analyzed for the consideration of long-term application in DMFC [50]. The mechanical strengths of the composite membranes are shown in Figure 7a. Clearly, all the curves show the same profile, the initial linear was typical elastic response, then the curve was the inelastic response before the yield point, and finally the curve is attained at the maximum strength at break [51,52,53]. The tensile strength of the composite membranes increases with the PVDF content in the composite membranes. The 60% PVDF membrane exhibits the best tensile strength (22 MPa), and the 30% PVDF membrane is the lowest of 17 MPa among the four composite membranes, which is comparable to that of Nafion 117 membrane (20 MPa) and might adequate for fuel cell operation application [54]. The typical thermal stability of the *s*-PI-PVDF composite membranes was collected under nitrogen atmosphere. The TG curves of the composite membranes are plotted in Figure 7b. The composite membranes show three-step weight loss thermograms in the curve analysis and can be comparable to the analogue aromatic *s*-PI [41]. The slight weight loss at about 170 °C was attributed to the evaporation of absorbed solvent and water. The second weight loss at about 400 °C was caused by the degradation of the sulfonic groups from the membrane. The degradation at about 500 °C is due to the decomposition of the main chain itself. The 5% mass loss temperature of the membrane is higher than 220 °C. In conclusion, the mechanical and thermal properties of the four *s*-PI-PVDF composite membranes are satisfied for DMFC applications. It can be concluded from the mechanical and thermal stability comparisons that the stability of the *s*-PI-PVDF composite membrane is strongly related to the PVDF content in the membrane. Enough PVDF in the membrane can provided continuous PVDF network for mechanical stability consideration and protection on the fragile segment of the *s*-PI polymer. Above all, all of the prepared *s*-PI-PVDF composite membranes meet the stability requirement of PEMs for applications in DMFCs (~80 °C) [2] and displays good thermal stability.

## 4. Conclusions

A hyper-branched sulfonated polyimide was designed and successfully synthesized as polymeric proton conductor. To prepare PEMs with high methanol-resistance and strong mechanical strength, PVDF was utilized as a binder to fabricate composite membranes with different content ratios. The detailed performances of these *s*-PI-PVDF composite membranes were measured by considering the requirements of DMFC application. The methanol permeability of these *s*-PI-PVDF composite membranes is revealed to be much higher than that of Nafion 117. All of the composite membranes also exhibit satisfied mechanical and thermal stabilities. With the good proton/methanol selectivity and great mechanical/thermal stabilities, the *s*-PI-PVDF composite membrane exhibited promising potential of application in DMFCs.

## Figures and Tables

**Table 1:** Water uptake, volume swelling and ion exchange capacity (*IEC*) of *s*-PI-PVDF composite membranes and Nafion 117.

Membranes	30% PVDF	40% PVDF	50% PVDF	60% PVDF	Nafion 117
Water uptake (%)	25.79 ± 0.03	17.87 ± 0.04	14.11 ± 0.04	9.05 ± 0.03	20.97 ± 0.05
Volume swelling (%)	26.95 ± 0.05	25.84 ± 0.06	24.73 ± 0.04	14.87 ± 0.04	36.29 ± 0.05
*IEC* (mmol/g)	0.6212 ± 0.03	0.5931 ± 0.02	0.5719 ± 0.03	0.3540 ± 0.04	0.91 ± 0.05
